# Construction and analysis for differentially expressed long non-coding RNAs and MicroRNAs mediated competing endogenous RNA network in colon cancer

**DOI:** 10.1371/journal.pone.0192494

**Published:** 2018-02-08

**Authors:** Fengxi Li, Qian Li, Xianghua Wu

**Affiliations:** 1 Department of Surgery, the First Affiliated Hospital of Guangxi Medical University, Nanning, Guangxi, China; 2 Department of Gynaecology and Obstetrics, the First Affiliated Hospital of Guangxi Medical University, Nanning, Guangxi, China; University of South Alabama Mitchell Cancer Institute, UNITED STATES

## Abstract

Long non-coding RNA (lncRNA) has been confirmed to act as a key regulatory molecule in different types of cancers and play a significant role in tumors initiation and progression. LncRNA can be as acompeting endogenous RNA(ceRNA) to regulate the expression of targeted genes by sponging miRNA. In the present study, we explore the functional roles and regulatory mechanisms of lncRNAs as ceRNAs in colon cancer and their potential implications for prognosis.The lncRNAs, miRNAs and mRNAs expression profiles of 341 colon cancer tissues and 27 non-tumor colon tissues were downloaded from The Cancer Genome Atlas (TCGA) database. Differential expression of RNAs was identified using the “DESeq” bioconductor package in R. PPI network of differentially expressed genes was constructed using the STRING database. Survival analysis was estimated based on Kaplan-Meier curve analysis. We used KOBAS 3.0 to analyze the KEGG pathway of DEGs. The dysregulated lncRNA-associated ceRNA network was constructed in colon cancer based on bioinformatics generated from miRanda, PicTar, TargetScan, miRDB and miRcode. A total of 791 DElncRNAs and 200 DEmiRNAs were identified in colon cancer compared with matched normal tissues with thresholds of |log2foldChange (FC)| >3.0and adjusted *P* value<0.05.Twenty DElncRNAs were identified, may be related to tumorigenesis and/or progression of colon cancer. Nine out of 20 dysregulated lncRNA were found to be significantly associated with overall survival (*P* value<0.05). Finally, we successfully constructed colon cancer-associated ceRNA network, including 9 colon cancer-specific lncRNAs, 13 miRNAS and 70 mRNAs. In conclusion, our study will contribute to improve the understanding of ceRNA network regulatory mechanisms in colon cancer. These identified novel lncRNAs can be as candidate prognostic biomarkers or potential therapeutic targets.

## Introduction

Colon cancer is one of the most prevalent malignancies in the world and it's the third leading causes of mortality worldwide[1]. In recent years, despite colon cancer diagnosis and treatment have developed rapidly. However, due to its high prevalence and poor prognosis, colon cancer is still an important clinical challenge worldwide. Therefore, identification of individualized treatment strategies, including potential biomarkers and therapeutic targets to combat colon cancer, is urgently needed.The present study explores how the colon cancer-specific lncRNAs act as ceRNA to regulate target genes and participate in pathogenesis and prognosis of colon cancer.

Research results show that more than 90% of the human genome sequence can be transcribed, but do not code for proteins[2]. LncRNA is a kind of non-coding RNA, with transcripts more than 200bp in length. At present, it has been identified and cloned more than fifty thousand in the human genome, but only a small part of the biological function of lncRNAs got the experimental verification and lncRNAs still remained poorly characterized. Dysregulations of lncRNAs are discovered in different types of tumors. These lncRNAs have significantpotential application prospect in the diagnosis, treatment and prognosis in malignant tumor[3]. However, lncRNA how to mediate the expression of genes has remained unclear. Considerable effort is being made to uncover that lncRNAs how to performance diverse biological functions in tumor. In 2011, Salmena et al. proposed a ceRNA hypothesis, which described a complex post-transcriptional regulatory network in which lncRNAs, mRNAs and other RNAs act as natural miRNA sponges to suppress miRNA function by sharing one or more miRNA response elements (MREs)[4]. LncRNA that harbors similar sequence to their targeted miRNAs functions as ceRNA regulates encoding protein gene level and participate in the regulation of cell biology by sponging miRNAs. Moreover, evidence is growing that lncRNA—miRNAs—mRNAs regulation networkiscrucialregulatory network is crucial regulatory molecules involved in pathogenesis and progression of tumors, including breast cancer, colon cancer, liver cancer, lung cancer and kidney cancer and other malignant tumors[5–10]. Studies have shown that lncRNAXIST was over-expressed in gastric cancer, which can be as a molecular sponge to modulate EZH2 expression by sponging miR-101 and it has been suggested possibly participating in the progression of gastric cancer[11]. Moreover, lncRNA PVT1 can influence the expression of HK2 by sponging miR-497[12]. We believe lncRNAs functions as ceRNAs deserve further exploration. In addition, the analysis of colon cancer associated lncRNA-mediated ceRNA network in a whole genome is lacking, especially studies with larger sample sizes.

By the end of 2015, TCGA collects more than 30 human tumor types and approximately 11,000 patient gene expression profiles together with relevant clinical data. Such an enormous amount of data provides a rich resource for data mining and to identify the mechanism and prognostic molecular signatures of cancer. In present research was based on TCGA, we analyzed the integrated RNA expression profiles from 341 colon cancer samples and 27 normal samples. As a result, 791 differentially expressed lncRNAs (DElncRNAs), 200 differentially expressed miRNAs (DEmiRNAs) and 1907 differentially expressed mRNAs (DEmRNAs) were identified in our study. In order to elucidate the interactions and valid potential crosstalk between RNAs, we successfully established the colon cancer associated ceRNA network based on bioinformatics generated from miRanda, PicTar, TargetScan, miRDB and miRcode, which included 9 lncRNAs, 13 miRNAs and 70mRNAs. Furthermore, relevant overall survival analyses were carried out to determine prognostic genes that possess clinical traits.

## Material and methods

### Study population

A total of 341 colon cancer cases were enrolled for comprehensively integrated analysis. The colon cancer level 3 RNAseq and miRNASeq of 341 colon cancer samples and corresponding clinical data (S1 File) were downloaded from TCGA database using the Data Transfer Tool (provided by GDC Apps)(https://tcga-data.nci.nih.gov/). The RNAseq and survival data were anonymised before we accessed them. These data were included 341 colon cancer samples and 27 non-cancer samples. That sequence data was derived from Illumina HiSeqRNASeq and Illumina HiSeqmiRNASeq platforms. Our research meets publication guidelines provided by TCGA (http://cancergenome.nih.gov/publications/publicationguidelines).

### Differentially expressed analysis

Colon cancer mRNAseq and miRNASeq data derived from 368 samples, which were classified into cohort Tumor (341 colon cancer samples) and cohort Normal (27 non-cancer samples), were downloaded from TCGA (S1 File). In addition, we combined tumor sample and non-cancer sample data and filtered out the expressed data which closed to zero. DEmRNAs/DEmiRNAs were identified in colon cancer samples compared with non-cancer samples using the DESeq, a Bioconductor package[13] in R software, with thresholds of |log2FC| >3.0 and adjusted P-value<0.05. We used GENCODE lncRNA annotation (V22), includes 15,877 human lncRNA genes encoding 26,414 transcripts (www.gencodegenes.org), to define and annotate the DElncRNAs.

### Construct the ceRNA network and functional annotation

In order to make clear the roles of lncRNA and miRNA with mediated ceRNA network, we built the co-expression network of lncRNAs-miRNAs-mRNAs interactions. MiRNA-targeted mRNAs were predicted by 4 programs, including miRanda, PicTar, TargetScan and miRDB. To improve accuracy, the most targeted genes were included in all the 4 datasets. To further improve the ceRNA network reliability, we retained mRNAs included in different expression of RNAs between tumor tissues and normal tissues. In addition, lncRNA-miRNA interactions were constructed based on miRcode database (http://www.mircode.org/). The ceRNA network was visualized using Cytoscape v3.5.0 software. Furthermore, KEGG pathways enrichment analysis of DEmRNAs was analyzed using KOBAS 3.0 (http://kobas.cbi.pku.edu.cn/)[14].

### Protein-protein interaction (PPI) networks construction

Inorder to better understand protein-protein interactions between differentially expressed genes (DEGs), we constructed PPI network for DEGs using the STRING (Search Tool for the Retrieval of Interacting Genes, https://string-db.org/) database (minimum required interaction score>0.4)[15]. Furthermore, PPI networks were embodied using the Cytoscape v3.5.0 software. In addition, we used Cytohubb plugin to identify the hub genes in PPI network. Top 10 hub genes were identified using the ranking method of degree.

### Survival analysis

In order to identify prognostic DERNAs signature, combining the clinical data of those patients with colon cancer in TCGA, we plotted the survival curves of those samples with DElncRNAs, DEmiRNAs and DEmRNAs by "survival" package in R. The univariate survival analysis was estimated based on Kaplan Meier curve analysis. *P-*value less than 0.05 was considered as statistical significance.

## Results

### Patient characteristics

A total of 341 patients were pathologically diagnosed as colon cancer. The median age of 341 colon cancer patients was 68 years old and forty-nine percent of them were more than 68 years old. Gender distribution: the male was more than female (male to female ratio: 1.08). Stage II and Stage III patients with colon cancer accounted for 67.7%. All 341 colon cancer patients with detailed pathological and clinical characteristics were shown in **Table 1**.

**Table 1:** Clinicopathological characteristics of 341 colon cancer patients.

Parameter	Subtype	Patients *n* (%)
Age (years)	>68	167 (49.0)
	≤68	174 (51.0)
Gender	Male	177 (51.9)
	Female	164 (48.1)
Race	WHITE	235 (68.9)
	ASIAN	12 (3.5)
	BLACK OR AFRICAN AMERICAN	66 (19.4)
	UNKNOW	28 (8.2)
Pathologic stage	StageI	54 (15.8)
	Stage II	131 (38.4)
	Stage III	99 (29.0)
	Stage IV	50 (14.7)
	UNKNOW	7 (2.1)
Pathologic T	T1	7 (2.1)
	T2	56 (16.4)
	T3	233 (68.3)
	T4	45 (13.2)
Pathologic N	N0	199 (58.4)
	N1	84 (24.6)
	NX^a^	58 (17.0)
Pathologic M	M0	245 (71.6)
	M1	50 (14.6)
	MX^a^	47 (13.8)

a: metastasis status unknown

### Differentially expressed mRNAs (DEmRNAs) in colon cancer

RNAs expression profiles of colon cancer patients and corresponding clinical information were downloaded using Data Transfer Tool of TCGA database. We identified the significant DEmRNAs and DEmiRNAs in colon cancer samples compared with the normal samples. A total of 1907 DEmRNAs were identified by DESeqpackage in R. As a result, there were 1476(77.40%) up-regulated and 431(22.60%) down-regulated DEmRNAs. A full list of DEmRNAs was shown **S1 Table**. The DEmRNAs was enriched in KEGG pathway by KOBAS 3.0 (http://kobas.cbi.pku.edu.cn/), in order to preliminary investigation tumorigenesis of colon cancer. These DEmRNAs were mainly enriched in “Wnt signaling pathway, Transcriptional misregulation in cancer and Chemical carcinogenesis”, which are closely correlated with tumor genesis (**Table 2**).

**Table 2:** DERNAs were enriched KEGG pathways in colon cancer.

Pathway ID	Description	P-value	Count
hsa04024	cAMP signaling pathway	0.023168	18
hsa04976	Bile secretion	2.27E-07	17
hsa00980	Metabolism of xenobiotics by cytochrome P450	6.31E-07	17
hsa05204	Chemical carcinogenesis	1.91E-06	17
hsa05202	Transcriptional misregulation in cance	1.30E-13	17
hsa00982	Drug metabolism–cytochrome P450	5.92E-06	15
hsa04974	Protein digestion and absorption	1.22E-04	15
hsa00830	Retinol metabolism	1.78E-05	14
hsa04970	Salivary secretion	3.58E-04	14
hsa04310	Wnt signaling pathway	0.022686	14

### Protein-protein interaction (PPI) networks construction

**Fig 1:**
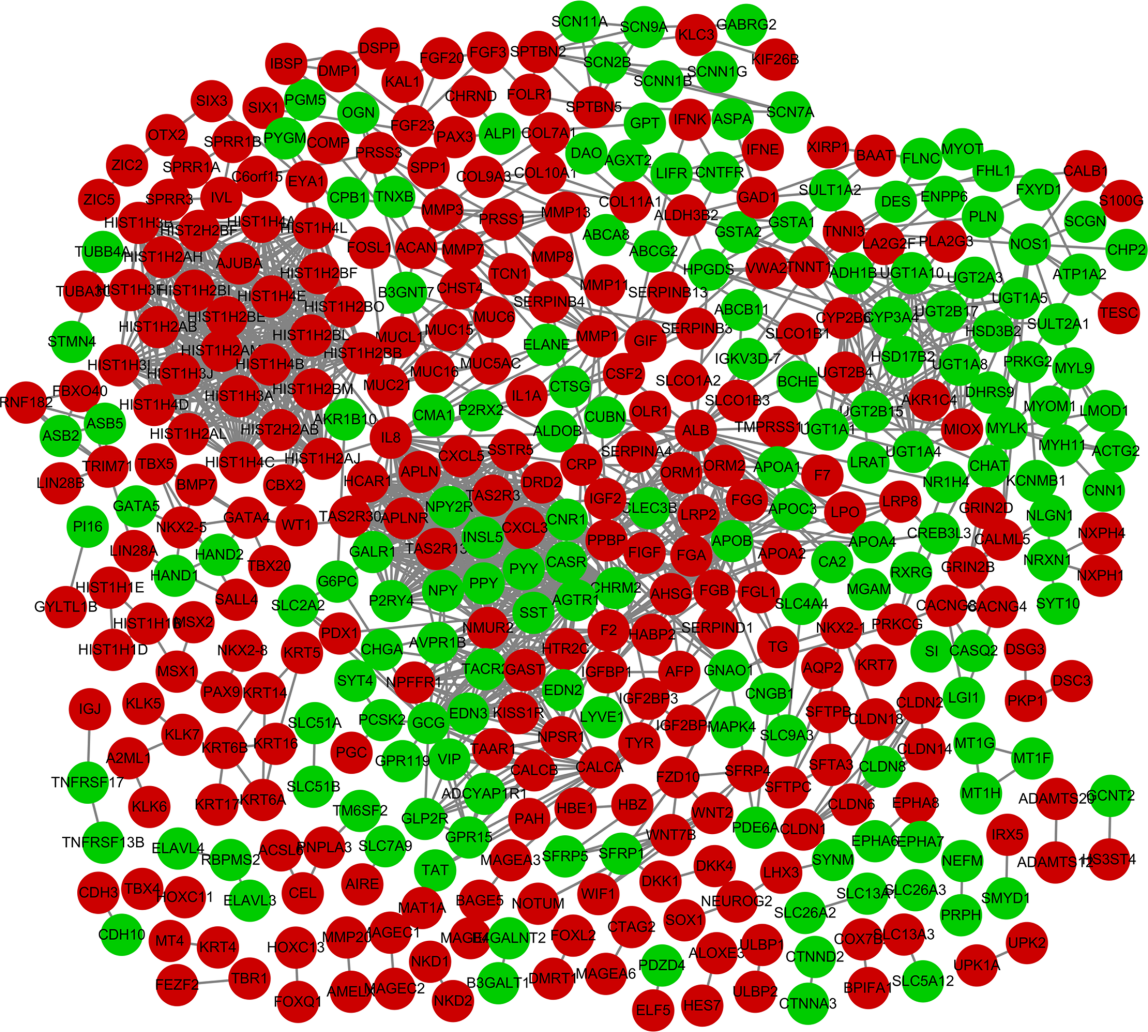
The PPI network of differentially expressed genes was constructed by STRING database in colon cancer. Each ellipse represents protein-coding gene. Down-regulated genes were green ellipse and up-regulated genes were red ellipse.

**Fig 2:**
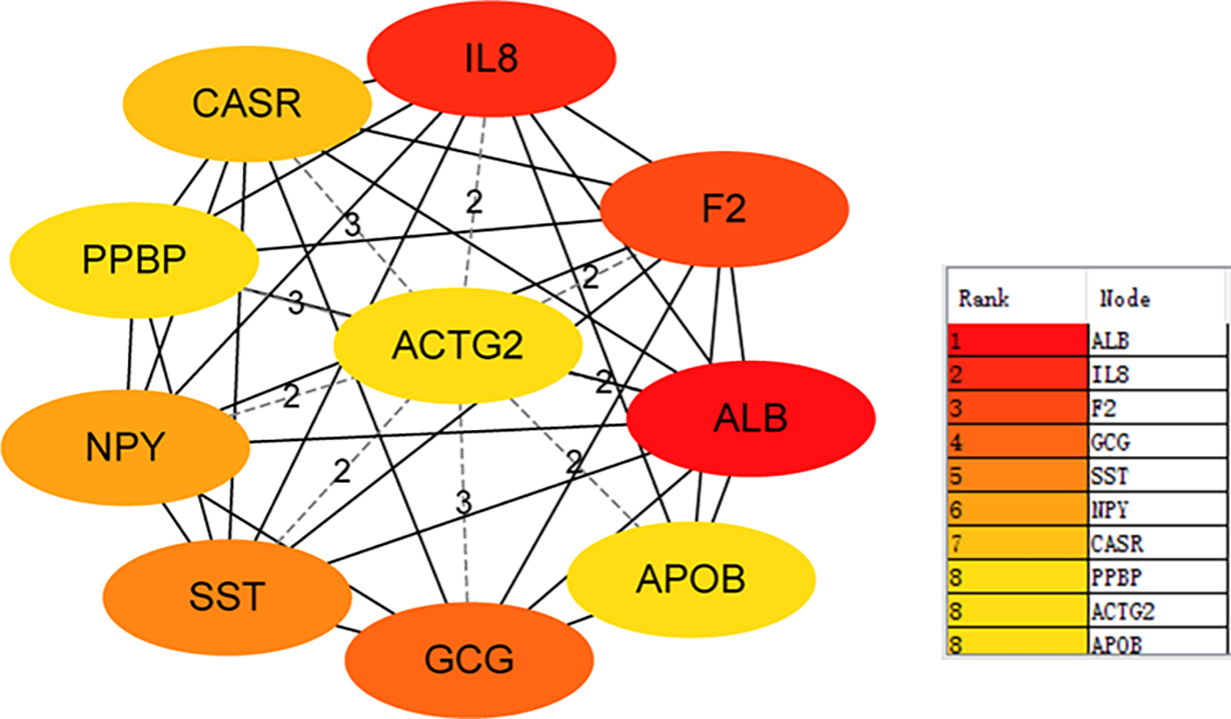
Top 10 hub DEGs were identified in colon cancer. Each ellipse represents gene.The solid lines between DEGs represent direct connections, and dashed lines represent indirect connections.

Furthermore, we used STRING database to excavate the interrelationship between DEGs by constructing PPI networks. The visualization of the PPI networks was embodied using the Cytoscape software (**Fig 1**). Moreover, we used Cytohubb plugin of Cytoscape to identify the hub genes in PPI network. We think the top 10 hub genes, ALB, F2, IL8, GCG, SST, NPY, PPBP, CASR, APOB and ACTG2, which may be closely related to colon cancer pathogenesis, using the ranking method of degree (**Fig 2**).

### Differentially expressed lncRNAs (DElncRNAs) in colon cancer

**Fig 3:**
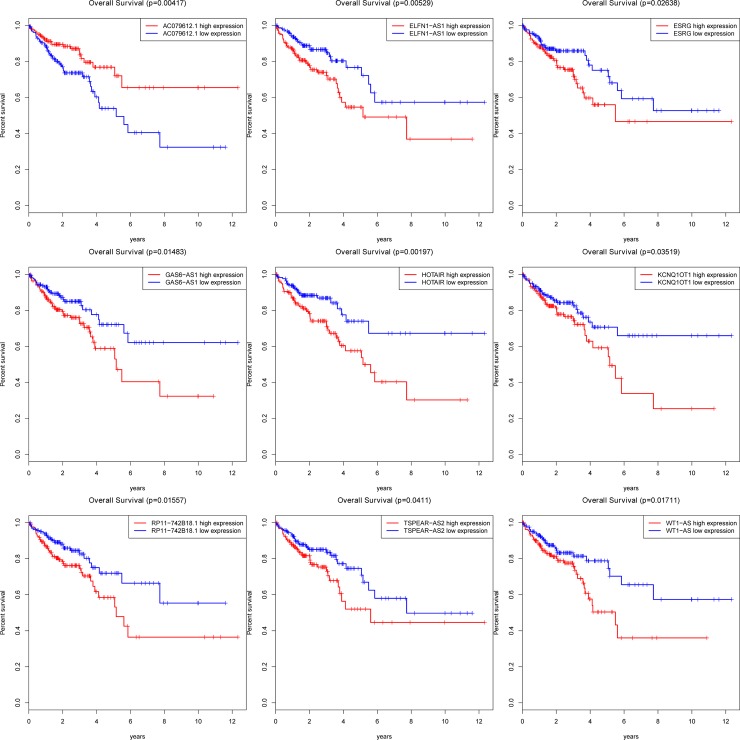
Nine lncRNAs associated with overall survival in colon cancer base on Kaplan-Meier survival curves. The horizontal axis represents overall survival time (years), vertical axis represents survival function. (9DElncRNAs were presented (P < 0.05), including KCNQ1OT1, WT1-AS, HOTAIR, GAS6-AS1, AC079612.1, ELFN1-AS1, RP11-742B18.1, TSPEAR-AS2 and ESRG).

A total of 791 DElncRNAs were identified in our study, with thresholds of |log2FC| >3.0 and adjusted P-value <0.05. A full list of DElncRNAs was shown **S2 Table**. To enhance the data reliability, those have not been annotated in GENCODE were removed. Finally, 12 DElncRNAs were identified in colon cancer samples compared to the normal samples (**Table 3**). Subsequently, to explore the relationship between DElncRNAs and the prognosis of patients with colon cancer, overall survival for 12 DElncRNAs in colon cancer patients was investigated using Kaplan-Meier curve analysis. We found that 9 of 12 DElncRNAs were considered as key DElncRNAs responsible for the prognosis of colon cancer. As a result, 9 DElncRNAs were significantly associated with overall survival, lncRNA AC079612.1 were positively correlated with overall survival, while the remaining 8 DElncRNAs were negatively associated with overall survival (log-rank P < 0.05)(**Fig 3**).

**Table 3:** Twelve colon cancer-specific lncRNAs in ceRNA network.

lncRNA	Gene ID	Expression change	log2 FC(T/N)	FDR
AC079612.1	ENSG00000196758	Up-regulation	3.61	1.74E-10
HOTAIR	ENSG00000228630	Up-regulation	3.13	0.00072
GAS6-AS1	ENSG00000233695	Up-regulation	3.53	5.71E-18
KCNQ1OT1	ENSG00000269821	Up-regulation	3.32	1.88E-09
RP11-742B18.1	ENSG00000249001	Up-regulation	4.58	2.69E-08
WT1-AS	ENSG00000183242	Up-regulation	3.46	3.64E-05
ERVH48-1	ENSG00000233056	Up-regulation	3.05	5.85E-05
MIR205HG	ENSG00000230937	Up-regulation	4.80	0.000855
LINC00400	ENSG00000229928	Up-regulation	4.75	0.001380
ELFN1-AS1	ENSG00000236081	Up-regulation	5.00	3.90E-33
TSPEAR-AS2	ENSG00000182912	Up-regulation	3.18	8.41E-10
ESRG	ENSG00000265992	Up-regulation	3.14	0.000263

Abbreviations: T: tumor; N: normal. FC: fold change

In addition, in order to further investigate lncRNAs took part in the progression of colon cancer. We also used “DESeq” package of R to identify the DElncRNAs (|log2FC|>3.0 and adjusted P-value<0.05). As a result, a total of 20 colon cancer specific lncRNAs were significantly different in the pathological tumor-node-metastasis (TNM) stages (Stage IV +Stage III vs. StageII+StageI, T4+T3 vs. T2+T1, N3+N2 vs. N1+N0, M1 vs. M0). We noted that 11 lncRNAs, SCARNA10, RPPH1, LINC01419, ERVH48-1, RMRP,CH507-513H4.3, MIR205HG, CH507-513H4.6, LINC00400, RP11-774D14.1 and AC006050.2, were also differentially expressed in colon cancer samples compared with the normal samples (**Table 4**). In addition, we found 6 out of 11 DElncRNAs, LINC01419, SCARNA10, RPPH1, LINC00400, RMRP and AC006050.2, were included in all the comparisons of pathological TNM stages. Therefore, we think these 6 DElncRNAs may be significantly associated with the tumorigenesis and/or progression of colon cancer.

**Table 4:** 11 lncRNAs associated with the tumorigenesis and /or progression of colon cancer.

Comparisons	Up-regulated	Down-regulated
Pathologic_Stage (Stage IV +Stage III vs. StageII+StageI)	LINC01419, LINC00400, RP11-774D14.1, AC006050.2	ERVH48-1, RPPH1, RMRP, SCARNA10, MIR205HG, CH507-513H4.6
Pathologic_T (T3+T4 vs. T1+T2)	LINC01419, LINC00400, RP11-774D14.1, AC006050.2	ERVH48-1, RPPH1, RMRP, MIR205HG, SCARNA10, CH507-513H4.6
Pathologic_N (N2+N3 vs. N0+N1)		SCARNA10, RPPH1, RMRP, LINC01419, LINC00400, AC006050.2
Pathologic_M (M1 vs. M0)	LINC01419, LINC00400 AC006050.2, RP11-774D14.1	RMRP, RPPH1, ERVH48-1 SCARNA10, CH507-513H4.6

### Differentially expressed miRNAs (DEmiRNAs) in colon cancer

In our study, 200 DEmiRNAs were identified in colon cancer samples compared with normal samples with thresholds of |log2FC| >3.0 and adjusted P-value<0.05(**S3 Table**). There are 146(73.00%) up-regulated and 54 (20.21%) down-regulated DEmiRNAs. A total of 18 DEmiRNAs (15 up-regulated and 3 down-regulated) were identified and maybe play an important role in pathogenesis of colon cancer (**Table 5**).

**Table 5:** Eighteen colon cancer specific miRNAs in ceRNA network.

Name	log2 FC (T/N)	*P*-value	FDR
hsa-mir-139	-4.39	4.26E-103	1.68E-100
hsa-mir-197	-4.04	8.25E-178	6.49E-175
hsa-mir-149	-3.34	2.22E-36	1.59E-34
hsa-mir-223	3.21	9.72E-08	7.05E-07
hsa-mir-337	3.26	7.39E-17	1.14E-15
hsa-mir-4709	3.78	0.005798	0.021126
hsa-mir-143	3.84	2.13E-08	1.63E-07
hsa-mir-17	3.87	3.78E-20	7.62E-19
hsa-mir-32	3.92	5.52E-23	1.45E-21
hsa-mir-429	4.23	9.77E-21	2.02E-19
hsa-mir-217	4.25	1.15E-08	9.14E-08
hsa-mir-182	5.31	1.75E-21	3.84E-20
hsa-mir-141	5.47	1.07E-38	8.44E-37
hsa-mir-96	5.52	7.09E-19	1.34E-17
hsa-mir-424	5.74	1.58E-24	4.79E-23
hsa-mir-144	6.17	8.40E-17	1.28E-15
hsa-mir-454	6.19	1.78E-23	4.96E-22
hsa-mir-653	7.95	2.01E-08	1.56E-07

Abbreviations: T: tumor; N: normal.

**Fig 4:**
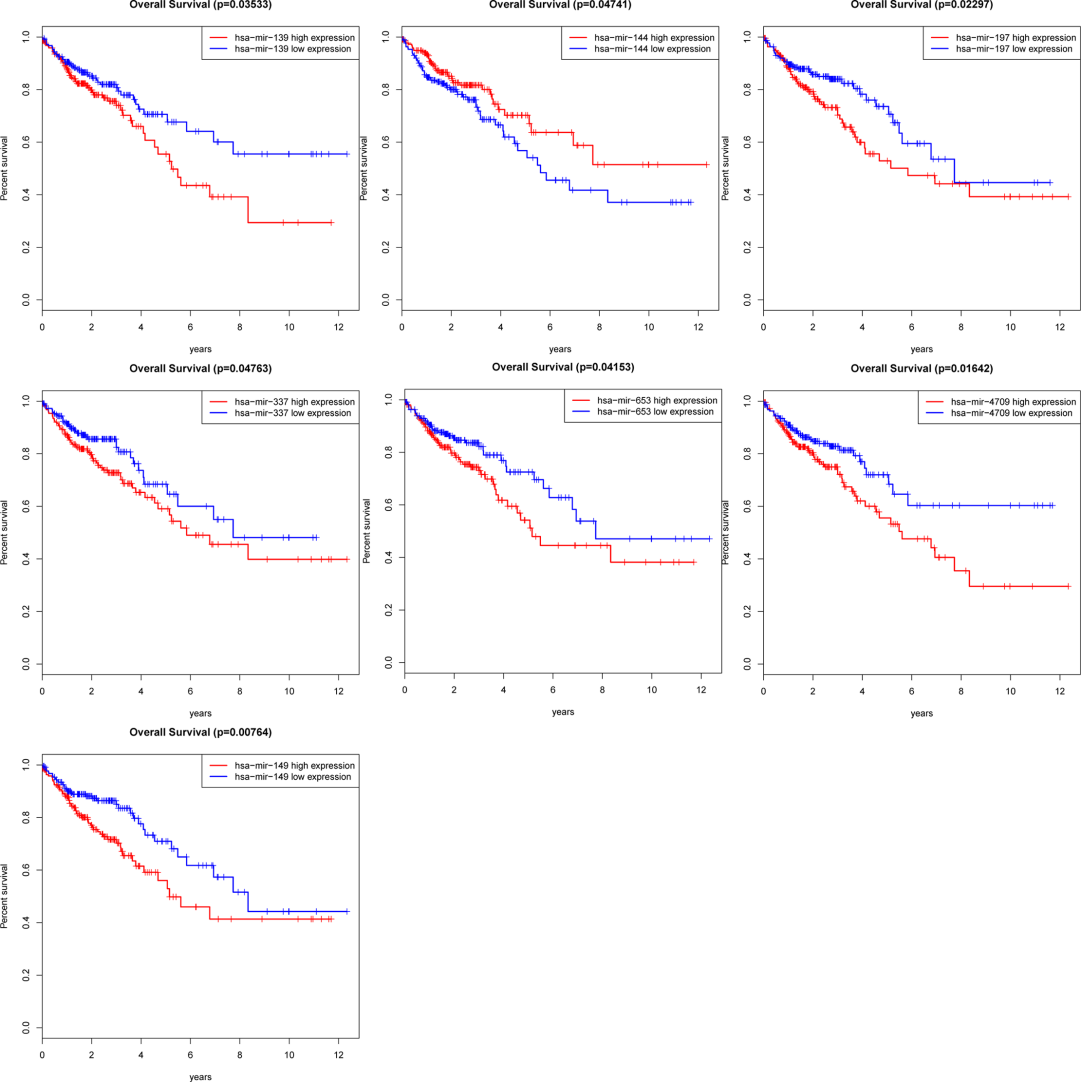
Four miRNAs associated with overall survival in colon cancer base on Kaplan-Meier survival curves. The horizontal axis represents overall survival time (years), vertical axis represents survival function. (7 DEmiRNAs were presented (P < 0.05), including miR-139, miR-144, miR-197, miR-337, miR-653, miR-4709 and miR-149).

As same with the DElncRNAs, the overall survival for 18 DEmiRNAs in colon cancer patients was also investigated using a Kaplan-Meier curve analysis. Seven out of 18 keys DEmiRNAs were significantly associated with overall survival (log-rank P < 0.05), and 6 DEmiRNAs, mir-139, mir-197, mir-337, mir-653, mir-4709 and mir-149 were demonstrated to be associated with high levels of DEmiRNAs and with poor prognosis. On the contrary, high levels of the remaining DEmiRNAs mir-144 were associated with prolonged patient survival time (**Fig 4**).

### ceRNA network in colon cancer

**Fig 5:**
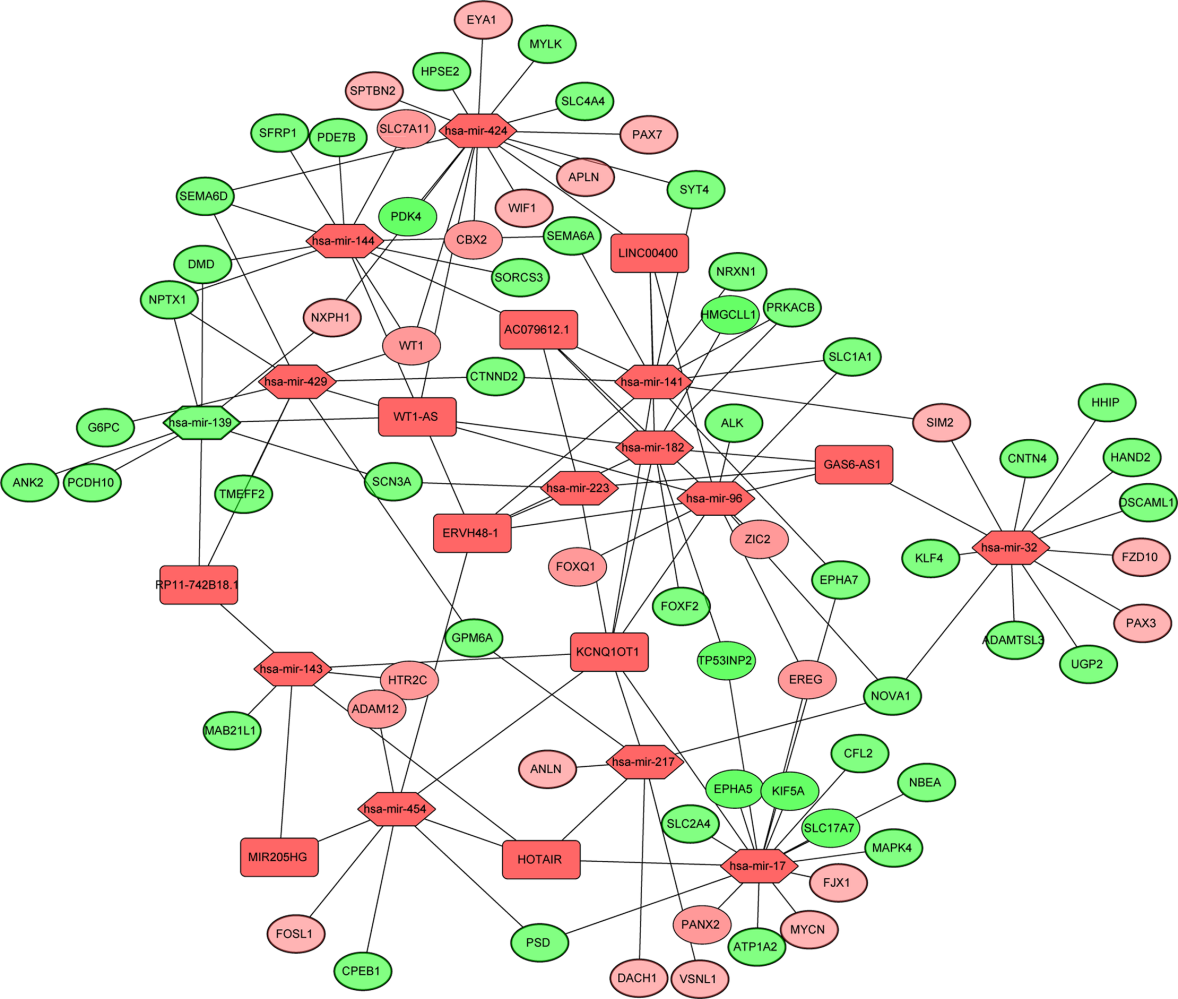
DElncRNAs-related ceRNA network in colon cancer. The red represent increased level of expression, while the green represent decreased level of expression. Round rectangle represent lncRNAs; hexagon represent miRNAs; ellipse represent protein-coding genes; black edges indicate lncRNA-miRNA-mRNA interactions.

To better understand how lncRNA mediate mRNA through combining miRNA in colon cancer, a ceRNA network graph was constructed based on above data and visualized using Cytoscape v3.5.0 (**Fig 5**). We found that 9 DElncRNAs could interact with the 13 DEmiRNAs retrieving miRcode database (**Table 6**).

**Table 6:** Nine DElncRNAs interact with the 13 DEmiRNAs retrieving miRcode database.

lncRNA	miRNAs
WT1-AS	miR-139, miR-429, miR-182, miR-96, miR-32, miR-17, miR-217, miR-141, miR-223, miR-424
KCNQ1OT1	miR-141, miR-143, miR-17, miR-182, miR-217, miR-223, miR-454, miR-96
GAS6-AS1	miR-182, miR-223, miR-32, miR-96
ERVH48-1	miR-141, miR-454, miR-182, miR-96, miR-223, miR-144
HOTAIR	miR-143, miR-17, miR-217, miR-454
RP11-742B18.1	miR-143, miR-429, miR-139
AC079612.1	miR-141,miR-182, miR-96, miR-223, miR-144
LINC00400	miR-96, miR-141, miR-182, miR-424
MIR205HG	miR-143, miR-454

**Fig 6:**
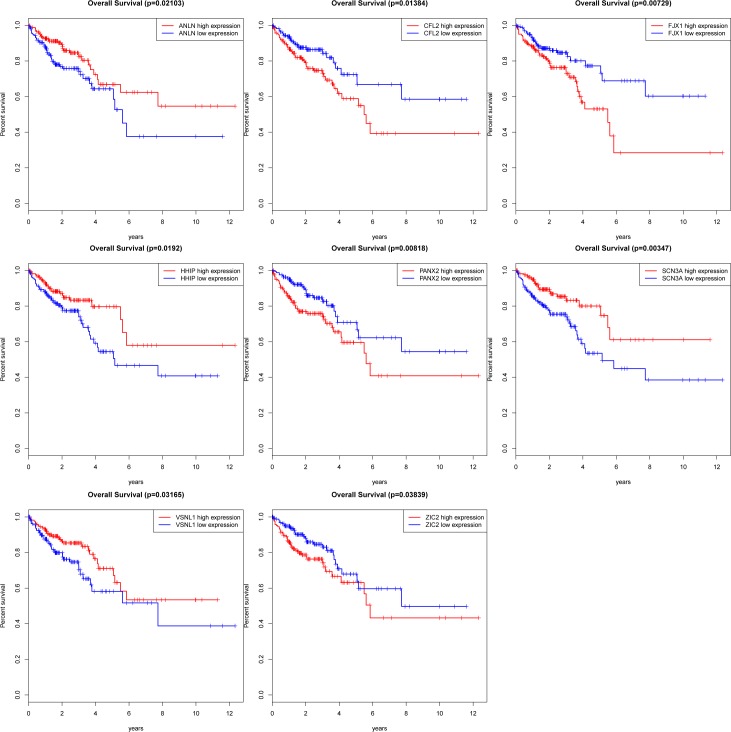
Eight protein-coding genes associated with overall survival in colon cancerbase on Kaplan-Meier survival curves. The horizontal axis represents overall survival time (years), vertical axis represents survival function. (8 DEmRNAs were presented (P < 0.05), including ANLN, CFL2, FJX1, HHIP, PANX2, SCN3A, VSNL1 and ZIC2).

We searched for targeted mRNAs based on 13 miRNAs using them iRanda, PicTar, TargetScan and miRDB database. The final targeted genes were selected, which most were included in all the 4 datasets. MiRNAs targeted mRNAs not included in DEmRNAs were discarded. Finally, 70 DEmRNAs were included in ceRNA network. We retrieved from the allOnco database (http://www.bushmanlab.org/links/genelists) found that most of them targeted genes involved in newly identified ceRNA network are definite tumor-related genes, such as WT1, MAPK4, MYCN, CNTN4, ADAMTSL3, KLF4, PAX3, ALK, EPHA7, ANLN, WIF1, PAX7, CTNND2, ADAM12 and FOSL1. Moreover, we noted that some of the mRNAs (ANLN, CFL2, FJX1, HHIP, PANX2, SCN3A, VSNL1 and ZIC2) were also associated with overall survival in patients with colon cancer (**Fig 6**).

Overall survival for DEmRNAs was also investigated base on Kaplan-Meier curve analysis. To better understand the KEGG pathways involved in ceRNA network, the mRNAs were performed using KEGG pathway analysis by KOBAS 3.0, and the top 6 KEGG pathways were significantly enriched cancer-associated signal pathways, such as “Transcriptional misregulation in cancer, Proteoglycans in cancer and Wnt signaling pathway” (**Table 7**).

**Table 7:** Top 6 KEEG pathways enriched by the coding genes involved in ceRNA network.

Pathway ID	Description	Genes
hsa04310	Wnt signaling pathway	PRKACB,FOSL1,FZD10,SFRP1,WIF1
hsa04360	Axon guidance	CFL2,EPHA5,SEMA6A,EPHA7,SEMA6D
hsa05202	Transcriptional misregulation in cancer	PAX3,PAX7,MYCN,EYA1
hsa04976	Bile secretion	PRKACB,SLC4A4,ATP1A2
hsa04971	Gastric acid secretion	PRKACB,ATP1A2,MYLK
hsa05205	Proteoglycans in cancer	ANK2,PRKACB,HPSE2,FZD10

**Fig 7:**
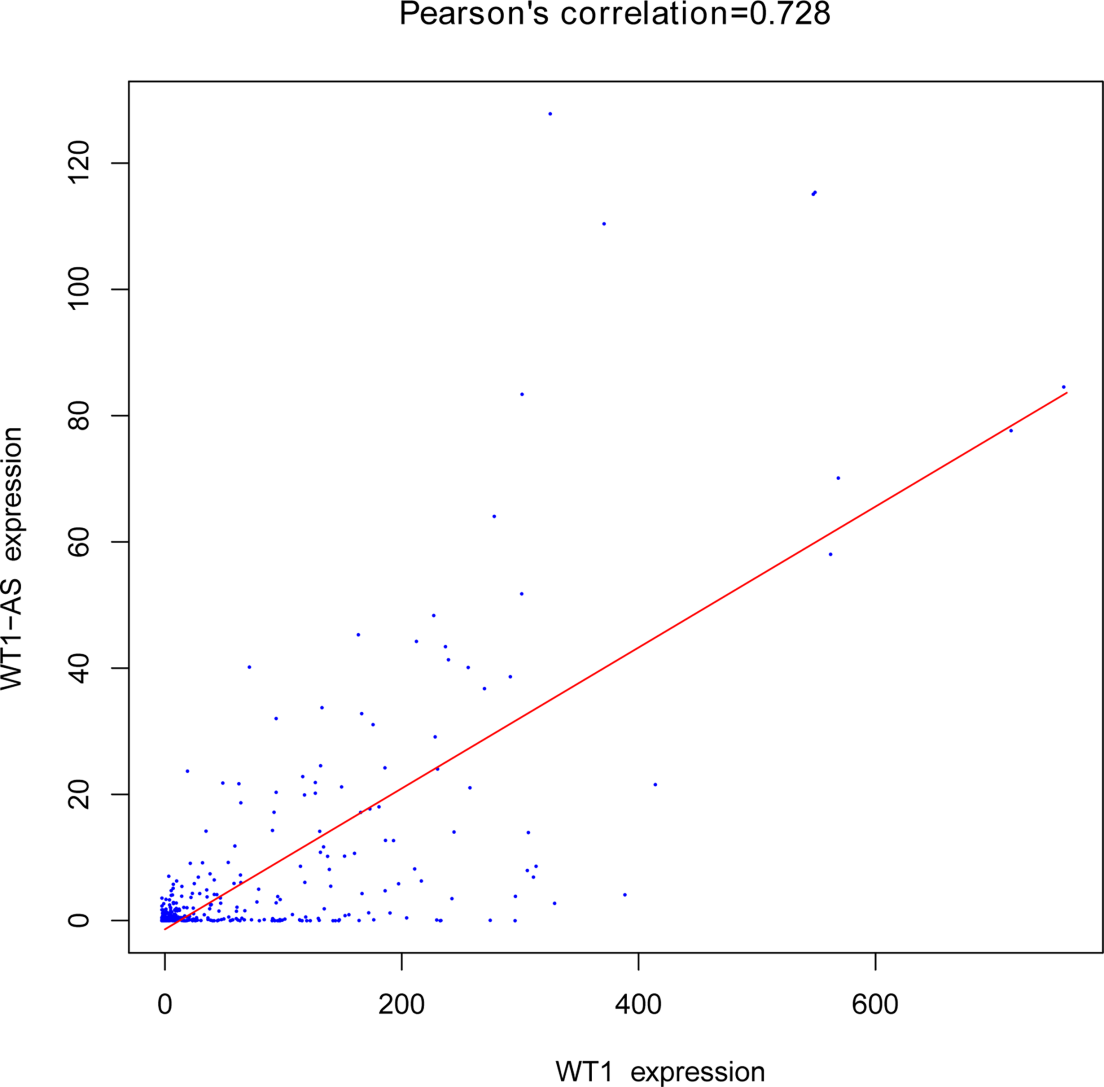
Regression analysis between the expression levels of WT1-AS and WT1 in ceRNA network. The horizontal axis represents WT1 expression, and vertical axis represents WT1-AS expression.

Moreover, the newly constructed ceRNA network maybe play an important role in colon cancer pathologenesis. Among ceRNA network, lncRNAs KCNQ1OT1 and WT1-AS maybe act as key lncRNAs, whichinteracted with 8 miRNAs (mir-143, mir-454, mir-96, mir-182, mir-17, mir-217, mir-141 and mir-223) and 5 miRNAs (mir-139, mir-429, mir-96, mir-182 and mir-424), respectively and indirectly interacted with 47and 33 miRNAs-targeted mRNAs in this network, respectively. In addition, we confirmed that the lncRNA WT1-AS was expressed in parallel with WT1 mRNA using a regression analysis (**Fig 7)**. In addition, we found that WT1 was miR-429 and miR-424 directly targeted gene retrieved from miRanda database. Therefore, we think WT1-AS can regulate the expression levels of gene WT1 by competing combined miRNA (miR-429 and miR-424), probably.

## Discussion

In 2012, there were 1.4 million new cases and 694,000 deaths from colorectal cancer. It is more prevalent in developed countries, where more than 65% of cases are found[16]. The occurrence of colorectal cancer is caused by many factors. The expression of abnormal gene expression is one of them, and the expression of molecular biological markers is considered to be related to the occurrence of colorectal cancer[17]. In recent years, many research revealed that DElncRNAs has important biological functions by regulating gene expression at various levels in multiple tumors, including epigenetic regulation, transcription regulation and post-transcription regulation[18, 19]. In addition, rising evidence showed that DElncRNAs present opportunity for different types of cancer diagnostics, prognostics and therapeutics[20]. Compared with protein-coding genes, lncRNAs have significant advantages as diagnostic and prognostic biomarkers[21]. A CeRNA hypothesis was proposed for the mechanism of tumorgenesis, providing an important new clue and the direction of research for tumor diagnosis and treatment, providing a new guiding theory[4]. It has been confirmed that lncRNAs that harbor similar sequence to their targeted miRNAs can sponge miRNAs to regulate mRNAs expression. Several studies have shown that theDElncRNAs is closely related to the pathogenesis and prognosis of tumor, and can be used as a tumor related predictors [22, 23]. In addition, research reported that up-regulation of lncRNA H19 significantly promotes epithelial to mesenchymal transition (EMT) progression and accelerates colorectal cancer cell growth by competing binding the miR-138 and miR-200a to modulate the derepression of targeted genes Vimentin, ZEB1, and ZEB2[24]. Peng et al. found lncRNA ROR can mediate the expression of miR-145 and participated in proliferation, migration and invasion of colon cancer cell[25]. Recent studies have revealed that the lncRNA PART-1 was up-regulated in colorectal cancer, functioned as a competitive endogenous RNA to regulate the targeted gene DNMT3A by sponging miR-143, participated in colorectal cancer cell proliferation and metastasis[26].

As far as we know, few researches have been performed to predict colon cancer prognosis.And there are few reliable colon cancer-specific lncRNAs as molecular biomarkers for risk stratification and detection of colon cancer. Therefore, we comprehensively integrated 341 colon cancer samples mRNA and miRNA data derived from TCGA and determined the lncRNAs-associated ceRNA network to further investigate the regulatory mechanism of lncRNAs.

In the present study, 12 DElncRNAs were identified in colon cancer samples compared with the normal samples. We found that 9 of them were significantly associated with overall survival, could be considered as a prognostic marker for colon cancer. Moreover, we noted that the lncRNA KCNQ1OT1 and WT1-AS were interacted with most of the miRNAs included in ceRNA network. Therefore, we think KCNQ1OT1 and WT1-AS may play a more significant role in the pathogenesis and prognosis of colon cancer. KCNQ1OT1 (KCNQ1 overlapping transcript 1) is a long non-coding RNA gene found in the KCNQ1 locus[27]. KCNQ1OT1 is a nuclear, 91 kb transcript, found in close proximity to the nucleolus in certain cell types[28]. It plays a major role in the transcriptional silencing of the KCNQ1 locus by regulating histone methylation[27]. The misregulation of the KCNQ1OT1 can lead to a variety of abnormalities. Over-expression of KCNQ1OT1 has been confirmed to be associated with many types of cancers, including wilms’ tumor, gliomas and colorectal cancers[29–31]. Consistent with previously reported, in our study, we identified that the lncRNA KCNQ1OT1 was up-regulated in 341 colon cancers compared with 27 non-tumor samples. A latest study shows that KCNQ1OT1 can be as a ceRNA to modulate miR-370 targeted gene CCNE2 by competing combined with miR-370[32]. Moreover, Xin et al. demonstrated that KCNQ1OT1 can impact expression levels of caspae-1 through directly interact with miR-214[31]. In our study, our analysis found that high expression of KCNQ1OT1 may compete with 8 key DEmiRNAs (miR-141, miR-143, miR-17, miR-182, miR-217, miR-223, miR-454 and miR-96) to mediate the expression of target genes. Moreover, patients with highly expressed KCNQ1OT1 had shorter survival time than those with low expression.

Furthermore, we consider WT1-AS may also play a key role in pathogenesis and prognosis of colon cancer. lncRNA WT1-AS is the antisense transcript of Wilms tumor 1 (WT1),which located upstream of WT1. WT1-AS is found on chromosome 11 and is expressed in kidney[33]. K. Moorwood et al. also found WT1-AS was expressed in kidney, positively regulated WT1 protein levels, and participated in the developing kidney[34]. Studies have shown that WT1-AS was expressed in parallel with WT1 mRNA. Moreover, several studies demonstrated WT1-AS may play an important role in the progression of ovarian cancer, acute myeloid leukemia and gastric cancer[35, 36]. In present research, WT1-AS was up-regulated in 341 colon cancer samples compared to 27 non-cancer samples. Survival analysis confirmed that high expression of WT1-AS was associated with poor prognosis in patient with colon cancer. Interesting, in our study, we performed a regression analysis between the expression levels of WT1-AS and WT1 targeted gene, which were both included in the newly constructed ceRNA network. The results revealed a very strong positive correlation between WT1-AS and WT1 expression levels. Furthermore, we noted that WT1 was miR-429 and miR-424 directly targeted gene retrieved from miRanda database. Based on the above results, we think that lncRNA WT1-AS may compete with 2 key DEmiRNAs (miR-429 and miR-424) to mediate the expression of WT1 target gene. However, these findings need further research to identify whether the lncRNA WT1-AS can be as a ceRNA to regulate the expression of gene WT1 by sponging miR-429 and miR-424.

Although lncRNA has received wide attentions in recent years, miRNAs also warrant increased attention. There is no doubt that tumorigenic-related pathways of research based on regulation of miRNAs are indispensable. Dysregulated expression miRNAs is reported to play various roles in carcinogenesis. In the present study, we identified tumor initiation-related miRNAs in colon cancer. Moreover, miR-139 and miR-144 not only involved in ceRNA network, but also closely associated with survival of colon cancer patient. MiR-139 aberration is found in wide variety types of human tumor, including gastric cancer, breast cancer, bladder cancer, hepatocellular carcinoma, ovarian cancer, colorectal cancer and so on. It has been confirmed that miR-139 was significantly down-regulated in colorectal cancer and play a crucial role in colorectal cancer metastasis and progression[37]. Consistent with previously reported, we confirmed miR-139 was down-regulated in 341 colon cancers compared with non-cancer samples. However,our survival analysis results indicated that the prognosis of colon cancer patients with high expression of miR-139 is poor. In addition, previous reports have revealed that the differential expression of miR-144 was associated with bladder cancer cell proliferation by regulating Wnt signaling pathway [38]. Moreover, Lwaya et al. [39]found miR-144 is related to the prognosis of colorectal cancer patients. In our study, survival analysis demonstrated that low expression of mir-144 leads to poor prognosis of colon cancer patients. However, those findings needed more research to identify whether those miRNAs have a specific role in tumorigenesis and prognosis of colon cancer.

The miRNAs-targeted genes involved in ceRNA network were performed KEGG pathways enrichment analysis results showed that targeted genes were mainly enriched Wnt signaling pathway, Transcriptional misregulation in cancer and Proteoglycans in cancer” cancer-associated pathways. These genes, SLC4A4, FOSL1, EYA1, FZD10, SFRP1, ATP1A2 and WIF1, not only enriched in KEGG pathway but also included in the ceRNA network. We found that these genes play a key regulatory role in the occurrence and development of tumors. The study found that FZD10 silencing can inhibit aberrant activation of Wnt3-FZD10-s. a specific role in tumorigenesis and prognosis of colon[40]. In addition, it has been confirmed that WIF1 (Wnt inhibitory factor-1), a Wnt pathway inhibitor, can inhibit human invasive urinary bladder cancer cells growth[41]. Interestingly, in our study, we also found that FZD10 and WIF1 were enriched in Wnt signaling pathway. Moreover, T Fukui et al. studies demonstrated that the SFRP1 gene is frequently downregulated and suppresses tumor growth activity of lung cancer cells[42]. They suggested that SFRP1 is a candidate tumor suppressor gene for lung cancer. Parks SK et al. found SLC4A4 plays a role in growth and migration of colon and breast cancer cells[43]. In addition, we noted that the targeted genes ANLN, CFL2, FJX1, HHIP, PANX2, SCN3A, VSNL1 and ZIC2, were also associated with overall survival of colon cancer patients (*P* value<0.05). In our study, we revealed how specific lncRNAs interact with miRNAs and coding genes through the successful construction of lncRNA—miRNAs—mRNA ceRNA network of colon cancer.

## Conclusion

In conclusion, 20 cancer-specific lncRNAs were identified from hundreds of candidate lncRNAs in large scale colon cancer samples. The research indicates that dysregulation of ceRNA network can lead to tumorigenesis[44]. We found that some lncRNA were remarkably associated with overall survival in patient with colon cancer. Importantly, we have successfully constructed a lncRNA-associated ceRNA network, which brings a new approach to lncRNA research in colon cancer and provides novel lncRNAs as candidate prognosis biomarkers or potential therapeutic targets.

## Supporting information

S1 Table A full list of DEmRNAs in colon cancer. (CSV)

S2 Table A full list of DElncRNAs in colon cancer. (CSV)

S3 Table A full list of DEmiRNAs in colon cancer. (CSV)

S1 File The minimal anonymized data set of colon cancer and non-cancer. (ZIP)
